# Cost-effectiveness of home care compared to hospital care in patients with chronic obstructive pulmonary disease (COPD): a systematic review

**DOI:** 10.3389/fmed.2024.1405840

**Published:** 2024-10-03

**Authors:** Maria Tereza Campos Vidigal, Guilherme Henrique Borges, Diogo Henrique Rabelo, Walbert de Andrade Vieira, Gustavo G. Nascimento, Rafael Rodrigues Lima, Márcio Magno Costa, Álex Moreira Herval, Luiz Renato Paranhos

**Affiliations:** ^1^Post-Graduate Program in Dentistry, School of Dentistry, Universidade Federal de Uberlândia, Uberlândia, MG, Brazil; ^2^Department of Dentistry, Centro Universitario das Faculdades Associadas de Ensino, São João da Boa Vista, SP, Brazil; ^3^National Dental Centre Singapore, National Dental Research Institute Singapore, Singapore, Singapore; ^4^Oral Health Academic Programme, Duke-NUS Medical School, Singapore, Singapore; ^5^Laboratory of Functional and Structural Biology, Institute of Biological Sciences, Universidade Federal do Pará, Belém, PA, Brazil; ^6^Department of Prosthodontics and Dental Materials, School of Dentistry, Universidade Federal de Uberlândia, Uberlândia, Brazil; ^7^Department of Preventive and Community Dentistry, School of Dentistry, Universidade Federal de Uberlândia, Uberlândia, MG, Brazil

**Keywords:** pulmonary disease, chronic obstructive, hospitals, Home Care Services, Hospital-Based Home Care Services, cost-effectiveness analysis

## Abstract

**Background:**

To compare, through a systematic literature review, the cost-effectiveness ratio of home care compared to hospital care for following up patients with chronic obstructive pulmonary disease (COPD).

**Methods:**

This review was registered in PROSPERO, and the bibliographic search was performed in six primary databases [MedLine (via PubMed), Scopus, LILACS, SciELO, Web of Science, and Embase], two dedicated databases for economic studies (NHS Economic Evaluation Database (NHS EED) and Cost-Effectiveness Analysis (CEA) Registry), and two databases for partially searching the “gray literature” (DansEasy and ProQuest). This review only included studies that compared home and hospital care for patients diagnosed with COPD, regardless of publication year or language. Two reviewers selected the studies, extracted the data, and assessed the risk of bias independently. A JBI tool was used for risk of bias assessment.

**Results and discussion:**

7,279 studies were found, of which 14 met the eligibility criteria. Only one study adequately met all items of the risk of bias assessment. Thirteen studies found lower costs and higher effectiveness for home care. Home care showed a better cost-effectiveness ratio than hospital care for COPD patients. Regarding effectiveness, there is no possibility of choosing a more effective care for COPD patients, given the incipience of the data presented on eligible studies. However, considering the analyzed data refer only to high-income countries, caution is required when extrapolating this conclusion to low- and low-middle-income countries.

**Systematic review registration:**

https://www.crd.york.ac.uk/prospero/, identifier CRD42022319488.

## 1 Introduction

Chronic obstructive pulmonary disease (COPD) affects the airways and other lung structures, and it is known for manifesting persistent respiratory symptoms and lung capacity limitations (1). It develops gradually and is related to a combination of different risk factors, such as exposure to active or passive smoking, chronic respiratory exposure to harmful products, recurrent respiratory infections, premature birth with interferences in lung growth, and even a genetic condition called alpha-1 antitrypsin deficiency (1, 2).

It is a highly prevalent condition, affecting 14% of men and almost 7% of women between 30 and 79 years old worldwide (3). Although COPD is treatable, it has high morbidity and mortality rates (2), and it was the third cause of global deaths in 2019, with more than three million deaths (1). Moreover, COPD patients are more vulnerable to developing mental disorders, such as depression and anxiety, as well as suicidal behaviors such as suicide ideation and attempts (4).

The treatment for COPD patients involves intensive care and recurrent or even continuous hospitalizations (5). Treatment also includes medication therapies and non-pharmacological interventions, such as pulmonary rehabilitation and oxygen therapy (6), added to expensive supplies, medications, and professionals (6, 7). Home care is an alternative form of health assistance for COPD patients (8, 9) and it is characterized by a set of prevention actions, disease treatments, rehabilitation, palliative care, and health promotion provided at home to ensure care continuity (10). This modality of care proposes to decrease the demand for hospital assistance (11), reduce the length of stay of hospitalized users (12), and optimize financial and structural resources (8).

Home care has shown good cost-effectiveness for following up individuals with other chronic diseases, such as cancer (13), chronic heart failure (14), coronary disease (15), chronic tuberculosis (16), and diabetes mellitus type 1 (17) and type 2 (15). Also, primary studies on COPD show that home care may be more inexpensive and effective than hospital care (8, 9, 18). Despite the existing studies assessing the healthcare cost-effectiveness ratio in COPD patients, robust evidence still lacks to support the decision-making of healthcare systems. Therefore, this systematic review evaluated the cost-effectiveness of home care compared to hospital care for following up COPD patients.

## 2 Materials and methods

### 2.1 Protocol registration and ethics consideration

All methods were carried out in accordance with relevant guidelines and regulations. The protocol of this systematic review followed the PRISMA-P (Preferred Reporting Items for Systematic Review and Meta-Analysis Protocols) guidelines (19) and was registered in the PROSPERO database^1^ (CRD42022319488). There were no changes or departures from the protocol when performing the review. The PRISMA (Preferred Reporting Items for Systematic Reviews and Meta-Analyzes) guidelines (20) and the JBI Manual for Evidence Synthesis (21) were used to report and conduct the method of this systematic review. Data were not collected from human participants for this systematic review. Ethical considerations of the primary studies included in this systematic review are presented in the results section.

### 2.2 Research question and eligibility criteria

This systematic review aimed to answer the guiding question, designed according to the PICO acronym (Population, Intervention, Comparator, and Outcome): For the treatment of COPD patients (P), is home care (I) more cost-effective (O) than hospital care (C)?

Inclusion criteria

• Population: Patients diagnosed with COPD, regardless of age, nationality, or disease stage;
• Intervention: Follow-up through home care;
• Comparator: Follow-up through hospital care;
• Outcome: Cost-effectiveness assessment at an individual level, regardless of the time horizon. The studies should report at least one finding related to the economic evaluation (healthcare costs, indirect medical costs, and costs outside healthcare) and intervention effectiveness (risk of death, readmissions, pulmonary functions, and quality of life);
• Study design: Randomized and non-randomized clinical trials.

Exclusion criteria

• Literature reviews, letters to the editor/editorials, personal opinions, books/book chapters, case reports/case series, pilot studies, preprint studies not yet submitted for peer reviewing, congress abstracts, and patents;
• Studies that did not clearly define the comparison group;
• Studies with oversampling (the most recent study that best described the methodology and results was considered in this case).

### 2.3 Sources of information and search

Electronic searches were performed up to February 2022 in the Embase, LILACS, MedLine (via PubMed), SciELO, Scopus, and Web of Science databases. Dedicated databases for economic studies–NHS Economic Evaluation Database (NHS EED) and Cost-Effectiveness (CEA) Registry–were also used. The DansEasy and ProQuest databases were searched to partially capture the “gray literature” and reduce the risk of publication bias. A search update was performed between February 2022 and August 2024. There were no restrictions on language or year of publication. The MeSH (Medical Subject Headings), DeCS (Health Sciences Descriptors), and Emtree (Embase Subject Headings) resources were used to select the search descriptors accordingly. Moreover, synonyms and free words composed the search. The Boolean operators “AND” and “OR” were used to improve the research strategy with several combinations. The search strategies in each database were made according to their respective syntax rules (Table 1). The results obtained in the primary databases were initially exported to the EndNote Web™ software (Thomson Reuters, Toronto, Canada) for cataloging and removing duplicates. The “gray literature” results were exported to Microsoft Word (Microsoft™, Ltd, Washington, USA) for excluding duplicates manually.

**TABLE 1:** Strategies for database search.

Database	Search strategy (February 2022, updated on August 2024)
**Main databases**	
Embase https://www.embase.com	#1 “chronic obstructive lung disease”/exp OR “chronic obstructive lung disease” OR “chronic disease”/exp OR “chronic disease” OR “lung disease”/exp OR “lung disease”
	#2 “home care”/exp OR “home care” OR “hospital discharge”/exp OR “hospital discharge”
	#3 “hospital care”/exp OR “hospital care” OR “hospital cost”/exp OR “hospital cost” OR “hospital”/exp OR “hospital” OR “hospitalization”/exp OR “hospitalization”
	#4 “cost benefit analysis”/exp OR “cost benefit analysis” OR “cost effectiveness analysis”/exp OR “cost effectiveness analysis” OR “healthcare cost”/exp OR “healthcare cost” OR “cost”/exp OR “cost”
LILACS http://lilacs.bvsalud.org/	#1 (MH: “Pulmonary Disease, Chronic Obstructive” OR COPD)
	#2 (MH: “Cost-Benefit Analysis” OR “Cost-Effective*” OR MH: “Healthcare Economics and Organizations” OR MH: “Costs and Cost Analysis” OR MH: “Healthcare Costs”)
PubMed http://www.ncbi.nlm.nih.gov/pubmed	#1 “Pulmonary Disease, Chronic Obstructive”[Mesh] OR COPD OR “Chronic Disease*”[Mesh] OR “Lung Disease*”[Mesh] OR “Lung Diseases, Obstructive”[Mesh]
	#2 “Home Care Services”[Mesh] OR “Home Care Agencies”[Mesh] OR “Home Nursing”[Mesh] OR “Home Health Nursing”[Mesh] OR “Home Treatment” OR “Hospital-At-Home” OR “Home Hospitalization” OR “Early Discharge” OR “Patient Discharge”[Mesh]
	#3 Hospital*[Mesh] OR “Hospital Care” OR “Health Centers” OR “Hospital Costs”[Mesh] OR “Hospitals, Chronic Disease”[Mesh] OR Hospitalization[Mesh] OR “Inpatient Hospital Care”
	#4 “Cost-Benefit Analysis”[Mesh] OR “Cost-Effective*” OR “Healthcare Economics and Organizations”[Mesh] OR “Fees and Charges”[Mesh] OR “Costs and Cost Analysis”[Mesh] OR “Healthcare Costs”[Mesh]
SciELO https://scielo.org/	#1 (“Pulmonary Disease, Chronic Obstructive” OR COPD)
	#2 (“Cost-Benefit Analysis” OR “Cost-Effective” OR “Healthcare Economics and Organizations” OR “Costs and Cost Analysis” OR “Healthcare Costs”)
Scopus http://www.scopus.com/	#1 TITLE-ABS-KEY (“Pulmonary Disease, Chronic Obstructive” OR COPD OR “Chronic Disease*” OR “Lung Disease*” OR “Lung Diseases, Obstructive”)
	#2 TITLE-ABS-KEY (“Home Care Services” OR “Home Care Agencies” OR “Home Nursing” OR “Home Health Nursing” OR “Home Treatment” OR “Hospital-At-Home” OR “Home Hospitalization” OR “Early Discharge” OR “Patient Discharge”)
	#3 TITLE-ABS-KEY (Hospital* OR “Hospital Care” OR “Health Centers” OR “Hospital Costs” OR “Hospitals, Chronic Disease” OR Hospitalization OR “Inpatient Hospital Care”)
	#4 TITLE-ABS-KEY (“Cost-Benefit Analysis” OR “Cost-Effective*” OR “Healthcare Economics and Organizations” OR “Fees and Charges” OR “Costs and Cost Analysis” OR “Healthcare Costs”)
Web of Science http://apps.webofknowledge.com/	#1 TS = (“Pulmonary Disease, Chronic Obstructive” OR COPD)
	#2 TS = (“Cost-Benefit Analysis” OR “Cost-Effective” OR “Healthcare Economics and Organizations” OR “Costs and Cost Analysis” OR “Healthcare Costs”)
**Dedicated databases**	
NHS Economic Evaluation Database (NHS EED) https://www.crd.york.ac.uk/CRDWeb/	#1 COPD
	#2 “Home Care”
	#3 “Hospital Care”
Global Health Cost-Effectiveness Analysis Registry (GH CEA) https://cevr.tuftsmedicalcenter.org/databases/cea-registry	#1 COPD
	#2 “Home Care”
	#3 “Hospital Care”
**Gray literature**	
DansEasy https://easy.dans.knaw.nl/ui/home	#1 (“Pulmonary Disease, Chronic Obstructive” OR COPD)
	#2 (“Cost-Benefit Analysis” OR “Cost-Effective” OR “Healthcare Economics and Organizations” OR “Costs and Cost Analysis” OR “Healthcare Costs”)
ProQuest https://about.proquest.com/en/dissertations/	#1 (“Pulmonary Disease, Chronic Obstructive” OR COPD OR “Chronic Disease*” OR “Lung Diseases” OR “Lung Diseases, Obstructive”)
	#2 (“Home Care Services” OR “Home Care Agencies” OR “Home Nursing” OR “Home Health Nursing” OR “Home Treatment” OR “Hospital-At-Home” OR “Home Hospitalization” OR “Early Discharge” OR “Patient Discharge”)
	#3 (Hospital OR “Hospital Care” OR “Health Centers” OR “Hospital Costs” OR “Hospitals, Chronic Disease” OR Hospitalization OR “Inpatient Hospital Care”)
	#4 (“Cost-Benefit Analysis” OR “Cost-Effective” OR “Healthcare Economics and Organizations” OR “Fees and Charges” OR “Costs and Cost Analysis” OR “Healthcare Costs”)

### 2.4 Study selection

After removing duplicates, the results were exported to Rayyan QCRI software (Qatar Computing Research Institute, Doha, Qatar) (22). Study titles were methodically analyzed (first phase) and those unrelated to the topic excluded. In the second phase, the abstracts of the studies were assessed with the initial application of the eligibility criteria. Titles that met the study objectives but without available abstracts were fully analyzed in the next phase. Subsequently, the eligible studies had their texts fully read to verify whether they met the eligibility criteria. If the full texts were not found, a bibliographic request was performed to the library database (COMUT), and an e-mail was sent to the corresponding authors to obtain the articles. Full texts published in languages other than English or Portuguese were translated to allow the application of the eligibility criteria. Two reviewers (MTCV and GHB), after previous calibration (Kappa = 0.87), independently performed all phases, and in case of doubt or disagreement, a third reviewer (LRP) was consulted to make a final decision.

### 2.5 Data collection

Before data extraction, to ensure consistency between the reviewers (MTCV and GHB), they performed a calibration exercise in which the data from one eligible study were extracted jointly. A third reviewer (LRP) conducted the calibration phase. Next, the full texts of the selected studies were reviewed, and the data were systematically extracted, including information on study identification (author, year, country, and location), sample characteristics (the number of participants included in each analysis group), collection and processing characteristics (description of home and hospital care methodologies, currency, and effectiveness variable), and main findings (specific costs from each group and effectiveness quantification). In case of incomplete or insufficient information, the corresponding author was contacted via e-mail.

An author (MTCV) extracted all the previous data and a second reviewer (GHB) performed a cross-examination to confirm the agreement among the extracted data. Any disagreement between the reviewers was solved by discussing it with a third reviewer (LRP).

### 2.6 Risk of bias assessment

Two authors (WAV and MTCV) independently assessed the risk of individual bias of the eligible studies with the JBI Critical Appraisal Tools for use in the JBI Critical Appraisal Checklist for Economic Evaluations (23). Each question could be answered as follows: “Yes” if the study did not have biases for the domain assessed in the question, “No” if the study had biases for the domain assessed in the question, “Uncertain” if the study did not provide sufficient information to assess the question biases, or “Not Applicable” if the question did not fit in the study. A third reviewer (LRP) was consulted in case of divergences between the evaluators.

### 2.7 Data synthesis

Considering the high heterogeneity of outcomes, measurement periods and methods, and health settings, the collected data were organized and described in a structured narrative synthesis according to the findings from each eligible study.

## 3 Results

### 3.1 Study selection

**FIGURE 1:**
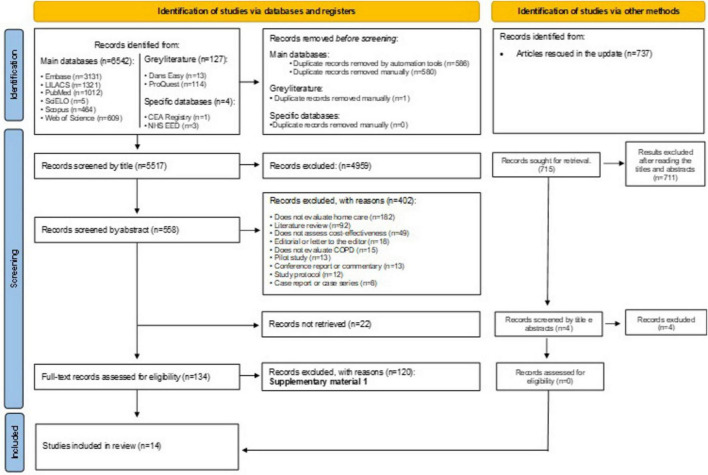
PRISMA flowchart.

In the first phase of study selection, 7,279 results were found distributed in ten electronic databases, including the economy-specific and “gray-literature” ones. After removing duplicates, 6,286 results remained for analysis. A careful reading of the titles excluded 5,459 results. Seven hundred and sixty-nine studies remained for abstract reading. Of these, 402 studies were excluded after applying the eligibility criteria, and 22 were not located even after applying different means of locating bibliographic records. The remaining 138 articles were fully read, of which 124 were excluded (Supplementary Appendix 1). Fourteen studies (8, 9, 11, 12, 18, 24–32) were included in the qualitative synthesis. Figure 1 displays the details of the search, identification, inclusion, and exclusion of studies.

### 3.2 Characteristics of eligible studies

The studies were published between 1988 and 2021 and performed in nine countries, with nine studies in Europe (8, 12, 18, 25, 27–29, 31, 32), two in Asia (9, 11), two in America (24, 30) and one in Oceania (26). Six studies (8, 12, 24, 30–32) did not mention to follow ethical criteria to conduct the research.

The total sample included 1,565 individuals with COPD subjected to home care and 7,932 under hospital care. Among the studies that reported the mean age of their sample, there was a variation between 63.6 (32) and 84.0 (29) years for the home care group and a variation between 61.2 (11) and 83.4 (30) years for the hospital care group. Regarding sex, there was a predominance of males (60.1 and 64.2%) among the studies that reported the sex of the participants for the home care and hospital care groups, respectively. Regarding the disease stage of the patients followed up through home care, six studies (12, 18, 26–28, 32) assessed patients in disease exacerbation (acute phase) and the remaining eight (8, 9, 11, 24, 25, 29–31) did not identify the disease stage and only informed it was a follow-up. The initial forced expiratory volume of the patients included in the studies ranged between 33.3 and 43% for patients under home care and 32.2 and 47% for those under hospital care. The application of both care options, such as follow-up time and the professional in charge and the tele-assistance technologies available to patients to contact the follow-up staff varied among studies (Table 2).

**TABLE 2:** Summary of the main characteristics of the eligible studies.

References (country)	Sample of home care (♀/♂)	Sample of hospital care (♀/♂	Mean age of home care	Mean age of hospital care	COPD stage	Applied home care	Applied hospital care	Mean FEV of home care (%)	Mean FEV of hospital care (%)
Bergner et al. (24) (United States)	77 (28/49)	86 (28/58)	65.2	64.3	Not specified	Respiratory home care program with nurses specially trained in respiratory diseases. Patients assigned to home nursing programs were seen by a home care nurse within 24 h of assignment and then as frequently as the nurse considered necessary, but at least once a month, during the study year.	In-person medical assistance, primary physician and care, and medications.	33.3	35.1
Shepperd et al. (25) (United Kingdom)	15 (10/5)	17 (14/3)	71	73	Not specified	The Rockingham Forest NHS Trust provided hospital home care with nursing, physiotherapy, occupational therapy, pathology, and speech therapy. Patients were provided with a mobile phone if required. Nursing was available 24 h a day in the patients’ homes if necessary.	Routine clinical practice.	nr	nr
Nicholson et al. (26) (Australia)	13 (nr)	12 (nr)	nr	nr	Exacerbation (acute)	The home care group was assisted by a hospital medical staff, the patient’s general practitioners, community nursing, and community allied health from the Domiciliary Allied Health Acute Care and Rehabilitation Team (DAART). The hospital medical staff provided 24-h telephone support and a “hot rescue referral” for trial patients who required readmission.	Routine clinical practice.	nr	nr
Puig-Junoy et al. (27) (Spain)	103 (2/101)	77 (2/75)	70.8	70.7	Exacerbation (acute)	The nurse scheduled the first home visit within 24 h after discharge. A maximum of five nurse visits at home were allowed during the 8-week follow-up period, but phone calls from patients to the nurse were unlimited.	Patients in the hospital care group were evaluated by the attending physician in the emergency room, who decided between inpatient hospital admission and discharge.	43	39.2
Aimonino et al. (28) (Italy)	39 (10/29)	41 (2/39)	80.1	79.2	Exacerbation (acute)	The trial was performed at the Geriatric Home Hospitalization Service (GHHS) and emphasized patient and caregiver education about the disease, advice about smoking cessation, nutrition, management of daily activities and energy conservation, knowledge and use of drugs, health maintenance, and early recognition of exacerbation triggers requiring medical intervention. In the first days after admission to GHHS, physicians and nurses visited each patient at home daily. In the following days, a nurse visited patients every day, and the doctor saw them at intervals of 2 to 3 days or less.	Routine clinical practice.	38	47
Bakerly et al. (18) (United Kingdom)	130 (77/53)	95 (21/74)	70	68	Exacerbation (acute)	Patients sent home with acute COPD assessment were visited by a specialist respiratory nurse until totally discharged. The nurse provided patients with short-term nebulizers and oxygen during exacerbation as per usual practice and was operational seven days a week, from 9:00 a.m. to 5:00 p.m.	Invasive ventilation of selected patients admitted to the hospital with acute exacerbation for 12 months.	nr	nr
Goossens et al. (12) (Netherlands)	70 (nr)	69 (nr)	68.3	67.8	Exacerbation (acute)	Patients randomized to early assisted discharge were sent home on the fourth day of admission and further treated at home. Community nurses visited the patient one to three times on the day of discharge and the three following days.	Usual non-pharmacological care consisted of physiotherapy for all patients for breathing and coughing instructions and dietary advice if indicated.	nr	nr
Vilá et al. (29) (Spain)	108 (nr)	18 (nr)	84	nr	Not specified	A nurse and an internist or geriatrician visited patients within 24 h after admission to the program. The home-care coordinating physician assisted individuals admitted to the hospital. As soon as they were stabilized, they were discharged to hospital home care.	Routine clinical practice.	nr	nr
Brian Cassel et al. (30) (United States)	65 (39/26)	189 (113/76)	82.8	83.4	Not specified	Concurrent home-based care program designed for individuals with advanced chronic illnesses who would benefit from the support of a trained specialized palliative care team comprising doctors, nurses, spiritual care providers, and social workers.	Routine clinical practice of Medicare Advantage beneficiaries served at medical groups affiliated with Sharp Healthcare.	nr	nr
Achelrod et al. (8) (Germany)	651 (241/410)	7,047 (2,537/4,510)	nr	nr	Not specified	Patients received up to two monitoring spirometers for mild to severe (FEV1 ≥ 35%) patients and a spirometer + pulse oximeter for very severe (FEV1 < 35%) patients to measure vital parameters at least twice a week. Patients were free to choose the time and day of vital parameter measurement.	Routine clinical practice.	≥ 70: 6.91 ≥ 50: 17.2 ≥ 35: 24.73 < 35: 39.63 Unknown: 11.52	≥ 70: 7.25 ≥ 50: 17.28 ≥ 35: 17.75 < 35: 25.20 Unknown: 32.51
Soriano et al. (31) (Spain)	115 (25/90)	114 (20/94)	71.5	71.3	Not specified	A nurse from the Monitoring Center enrolled patients in the program by registering them in a dedicated data management portal and scheduling a home visit. That was performed by healthcare personnel who installed monitoring equipment and trained the patient or caregiver on its use.	Routine clinical practice.	34.2	32.2
Widyastuti et al. (11) (Indonesia)	18 (2/16)	18 (3/15)	68.3	61.2	Not specified	Home pedometer assisted physical activity, and the patient should walk at home for six weeks, as fast as possible, for at least 30 min every day. There were weekly meetings at the patients’ homes. They were instructed to record the number of daily steps in activity logbooks and information on any changes in clinical conditions.	Patients received three 30-min weekly sessions for six weeks of supervised standard exercise training on a treadmill at outpatient clinics.	< 80: 17 50–79: 44 30–49: 33 < 30: 6	< 80: 11 50–79: 50 30–49: 22 < 30: 17
Duiverman et al. (32) (Netherlands)	33 (18/15)	34 (22/12)	63.6	63.1	Exacerbation (acute)	Home care patients were initiated on chronic non-invasive ventilation exclusively at home, using telemedicine. Ventilator data were retrieved via a GPRS system clicked on the back of the ventilator, which sent data to an online platform. Changes in ventilator settings could be made remotely. The specialized nurse visited the patient on day 1 to install the equipment, explain all procedures, and practice with NIV.	In-hospital ventilation according to the regular procedures of the pulmonary ward.	nr	nr
Jacobs et al. (9) (Israel)	10 (nr)	13 (nr)	73.8	73.4	Not specified	Prolonged invasive mechanical ventilation at home included emergency 24-h on-call visits from a respiratory technician. Home caregivers received training and instructions from the home care hospital staff to guarantee competence in basic skills to ensure adequate long-term ventilator care, such as providing inhalation and suction and immediate first aid if the cannula was accidentally removed.	Hospital long-term care.	nr	nr

nr, not reported in the study; FEV, forced expiratory volume.

As for cost analysis, the currencies used in the studies were US dollar (24, 26, 28, 30), euro (8, 11, 12, 27, 29, 32), sterling pound (18, 25), and Israeli shekel (9). Moreover, different economic evaluation perspectives were addressed, between them societal perspective (12, 24, 25, 32), patient perspective (18, 25, 28), healthcare and services perspective (9, 11, 12, 26, 29–31), public insurer perspective (27), and sickness fund perspective (8).

Regarding the effectiveness analysis, the following factors composed the indicators: hospital readmission rates, exacerbation rates, the number of visits to the hospital emergency sector, and the number of deaths. One study (11) assessed home care follow-up related to respiratory exercises. In that case, effectiveness was analyzed with body mass and exercise indices and airflow obstruction and dyspnea rates.

### 3.3 Risk of individual bias in the studies

**FIGURE 2:**
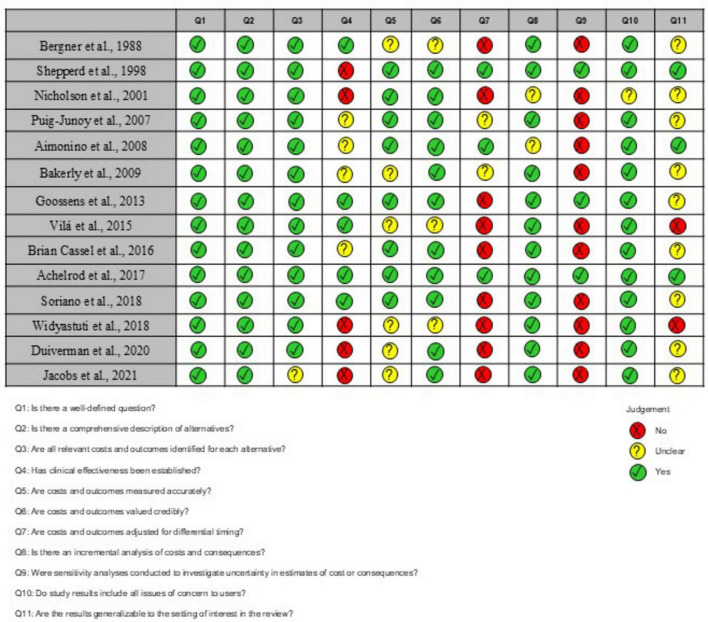
Risk of bias assessed with the Joanna Briggs Institute Critical Appraisal Tools for use in the JBI Critical Appraisal Checklist for economic evaluations.

Only one study (8) presented positive answers to all tool items. Question 4 (Has clinical effectiveness been established?) received only five positive responses because most eligible studies did not specify clinical efficacy or the origin of efficacy estimation. That might increase the risk of bias because there was no confirmation of a solid evidence base supporting the assumptions about the direction and magnitude of the efficacy measurement. Question 5 (Are costs and outcomes measured accurately?) received six unclear answers, indicating that cost and outcome measurement precision was incorrectly reported. Questions 9 (Were sensitivity analyses conducted to investigate uncertainty in estimates of cost or consequences?) and 11 (Are the results generalizable to the setting of interest in the review?) received only three positive answers because numerous studies did not perform a sensitivity analysis to investigate the uncertainty in cost estimates and did not obtain results generalizable to other environments, respectively. Figure 2 provides detailed information on the risk of bias in the eligible studies.

### 3.4 Specific results of the eligible studies

**FIGURE 3:**
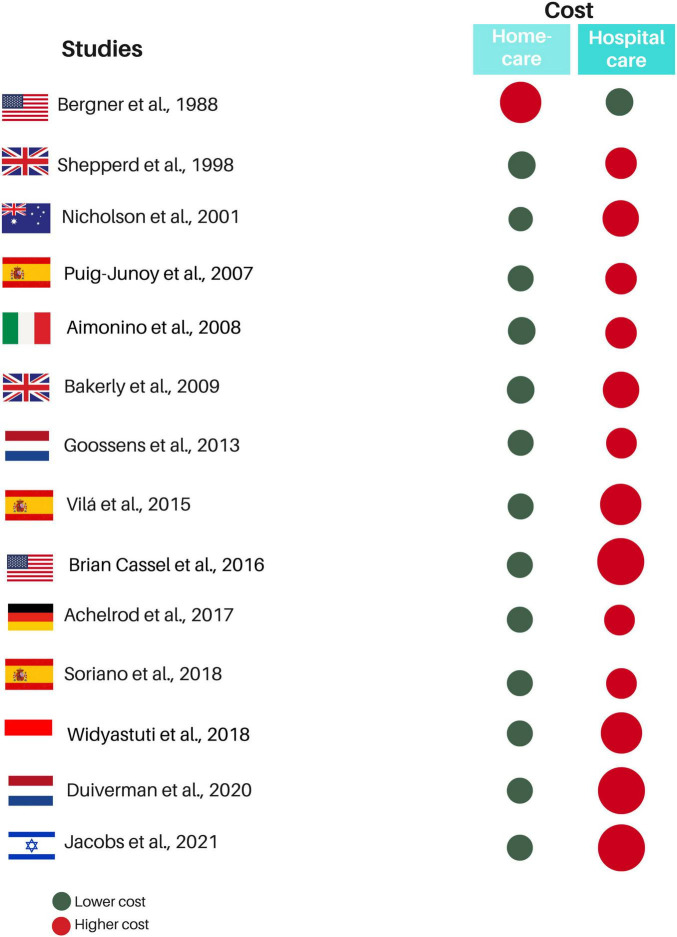
Summary of quantitative results in eligible studies. Red circles represent the treatment with the highest cost, while the green circle represents the treatment with the lowest cost. The size difference between the red and green circles within the same study indicates the proportional cost difference between the treatments. The greater the cost difference, the larger the red circle.

Only one study (25) reported the discount rate percentage, and two others (8, 28) informed applying the discount but not the rate. Total costs varied, and this variation referred to the type and services included in the provided home care. Home care costs were lower in the follow-up cases of patients with COPD exacerbation, which is the acute phase of the condition (12, 18, 26–28, 32). For investigations that did not specify the COPD phase, costs were also lower for home care, except for the study by Bergner et al. (24), which showed charges of 8,085 dollars for standard home care and 9,767 dollars for specialized home care for respiratory diseases, while hospital care cost 5,051 dollars (Figure 3). Only five studies (8, 11, 12, 18, 27) calculated incremental costs. Table 3 shows the cost analysis information of the included studies.

**TABLE 3:** Main results and outcomes of the eligible studies regarding cost analysis.

References	Perspective	Time horizon and currency year	Discount rate	Comparator/ Intervention	Total costs	Incremental costs
Bergner et al. (24)	Societal	12 months Dollar ($) 1981	nr	Hospital care/Standard home care and specialized respiratory home care with physicians	5,051 $/8,058 $ and 9,767 $	nr
Shepperd et al. (25)	Societal and Patient	3 months Pound (£) 1995	6%/year	Inpatient hospital care/Hospital home care	To social services: 3,292 £/2,516 £ To patients: 77 £/60 £	nr
Nicholson et al. (26)	Health services	nr Dollar ($) 2000	nr	Traditional hospital care/Integrated home care for acute COPD	Per episode: 2,543 $/745 $	nr
Puig-Junoy et al. (27)	Public insurer	12 months Euro (€) 2000	nr	Conventional hospitalization/Home hospitalization for COPD exacerbation	Per patient: 1,964 €/1,154 €	nr/647 €
Aimonino et al. (28)	Patient	6 months Dollar ($) 2005	Referring to the currency year	General medical ward/Geriatric home hospitalization for acute COPD	Per patient per day: 152 $/101 $	nr
Bakerly et al. (18)	Patient	6 months Pound (£) 2007	nr	Hospital treatment/Integrated care with home treatment for acute COPD	Per patient: 2,256 £/1,653 £	nr/600 £
Goossens et al. (12)	Heath care and Societal	3 months Euro (€) 2011	nr	Inpatient hospital treatment/Home treatment under early assisted discharge	Per day: 1,430 € / 976 €	nr/454 €
Vilá et al. (29)	Health services	6 months Euro (€) 2013	nr	Hospital Care/Home care program	Per person per day: 73 €/24 €	nr
Brian Cassel et al. (30)	Health services	18 months Dollar ($) 2014	nr	Clinic-based/Home-based palliative care	Per month: 5,859 $/1,697 $	nr
Achelrod et al. (8)	Sickness fund	12 months Euro (€) 2013	Referring to the currency year	Hospital standard care group/Home telemonitoring	9,371 €/8,314 €	nr/177.7 €
Soriano et al. (31)	Health services	12 months Euro (€) nr	nr	Routine clinical practice/Telehealth	8,918 €/7,912 €	nr
Widyastuti et al. (11)	Health services	6 weeks Euro (€) 2017	nr	Standard supervised PA/Home pedometer assisted PA	123.7 €/53.7 €	nr/76.3 €
Duiverman et al. (32)	Societal	6 months Euro (€) 2017	nr	Hospital NIV/Home NIV with telemedicine	8,537 €/3,768 €	nr
Jacobs et al. (9)	Health services	1 month New Israeli Shekel ( 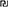 ) 2018	nr	Hospital long-term care/Home PMV	45,000 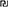 /15,000 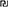	nr

nr, not reported in the study; PA, physical activity; NIV, non-invasive ventilation; PMV, prolonged mechanical ventilation.

Among the few studies that reported indicators to assess effectiveness, the following were applied: QALY (quality-adjusted life years) (12) and YLL (years of life lost) (8) (Table 4). The readmission rate in the emergency sector was lower for patients followed up at home (28, 30). Finally, three studies (8, 12, 25) performed sensitivity analyses, ranging from two to six analysis scenarios.

**TABLE 4:** Main results and outcomes of the eligible studies regarding the effectiveness analysis.

References	Perspective	Comparator/Intervention	QALYs/DALYs/YLL	Incremental effectiveness	ICER/INMB	CEACs*	Sensitivity analysis
Bergner et al. (24)	Societal	Hospital care/Standard home care and specialized respiratory home care with physicians	nr	nr	nr	Nr	nr
Shepperd et al. (25)	Societal and Patient	Inpatient hospital care/Hospital home care	nr	nr	nr	Nr	Assuming inpatient costs at 75 or 50% of the baseline
Nicholson et al. (26)	Health services	Traditional hospital care/Integrated home care for acute COPD	nr	nr	3:1	Nr	nr
Puig-Junoy et al. (27)	Public insurer	Conventional hospitalization/Home hospitalization for COPD exacerbation	nr	nr	nr	Nr	nr
Aimonino et al. (28)	Patient	General medical ward/Geriatric home hospitalization for acute COPD	nr	nr	nr	nr	nr
Bakerly et al. (18)	Patient	Hospital treatment/Integrated care with home treatment for acute COPD	nr	nr	nr	nr	nr
Goossens et al. (12)	Heath care and Societal	Inpatient hospital treatment/Home treatment under early assisted discharge	QALYs: 0.175/0.170	QALY: 31.111	Base case: 31.111	nr	With six scenarios
Vilá et al. (29)	Health services	Hospital care/Home care program	Nr	nr	nr	nr	nr
Brian Cassel et al. (30)	Health services	Clinic-based/Home-based palliative care	nr	nr	nr	nr	nr
Achelrod et al. (8)	Sickness fund	Hospital standard care group/Home telemonitoring	YLL: nr/YLL: 108,689/year	nr	nr/191 € per QALY	nr	With three scenarios
Soriano et al. (31)	Health services	Routine clinical practice/Telehealth	nr	nr	nr	nr	nr
Widyastuti et al. (11)	Health services	Standard supervised PA/Home pedometer assisted PA	nr	nr	nr	nr	nr
Duiverman et al. (32)	Societal	Hospital NIV/Home NIV with telemedicine	nr	nr	nr	nr	nr
Jacobs et al. (9)	Health services	Hospital long-term care/Home PMV	nr	nr	nr	nr	nr

QALYs, quality-adjusted life years; DALYs, disability-adjusted life years; YLL, years of life lost; ICER, incremental cost-effectiveness ratio; INMB, incremental net monetary benefit; CEACs, cost-effectiveness acceptability curves; nr, not reported in the study; PA, physical activity; NIV, non-invasive ventilation; PMV, prolonged mechanical ventilation;

*The monetary values are each “threshold” and the percentages are the cost-effectiveness probability for the specific outcome measure.

## 4 Discussion

This systematic review investigated the scientific literature to assess whether home care has a better cost-effectiveness ratio for following up COPD patients than hospital care. The synthesis showed that home care usually has lower costs and higher effectiveness for following up on COPD patients.

Among the main challenges of health systems, adequate management of resources to optimize the processes involving healthcare coverage and access for the population stand out. Patients who require intensive care and recurrent or even continuous hospitalizations, such as COPD patients, may bear higher costs with supplies, medications, and professionals (5). In pandemic times, such as COVID-19 or other disease outbreaks, the search for hospital beds in outpatient or intensive care increased abruptly (33, 34). Moreover, overcrowding has also contributed to a higher risk of hospital contamination or infections (34), especially concerning immunocompromised individuals, such as COPD patients under long-term pharmacological treatment with immunosuppressants (35). Hence, home care represents a relevant alternative to the healthcare of COPD patients.

Home care for following up COPD patients may be applied differently, with potential variations in the service time, expertise of professionals in charge of follow-up, and tele-assistance technologies available to patients to contact the staff. The data of the eligible studies regarding these factors confirm these variations, but regardless of them, it was almost unanimous that home care reduced costs. Only Bergner et al. (24) reported a higher charge for home care than hospital care. This difference from the other eligible studies may be due to the incipient technological development and the high cost of information and communication media at the time of the study, considering that patients were followed up with the help of tele-assistance in the late 80s, when such a technology was more restricted and, consequently, expensive.

Regarding the effectiveness analysis among eligible studies, one fact drew attention: even though they claimed to have performed cost-effectiveness analyses, the cost data were generally more complete, while the data regarding effectiveness were mostly incipient. Only two studies (8, 12) applied effectiveness analysis indicators and provided results on this analysis, and these results did not present a statistically significant difference. Even though they performed narrative descriptions of some effectiveness factors, such as hospital readmissions and lung functions, an effectiveness analysis with known and widely used indicators is extremely important to reveal more valuable effectiveness results. Therefore, it is not feasible to indicate better results for one of the groups.

Besides the variations in the type of home care used, COPD has heterogeneous clinical characteristics manifested with different symptom severity levels at each disease stage (36, 37). Considering this assumption, only six eligible studies reported including only COPD patients in exacerbation, which is an acute phase of the disease (12, 18, 26–28, 32). Acute COPD patients usually manifest exacerbated symptomatology (27), which may interfere with the required care and costs. The other studies did not specify the COPD stage or the clinical condition of patients when performing the analyses. Considering that COPD is a complex disease, it would be crucial to analyze these differences in further studies to obtain homogeneous evidence according to the clinical condition of patients.

In this review, all studies were performed in high-income countries. However, the availability of specialized professionals and funding and the offer of tele-assistance technology differs between high- and low-middle-income countries (38), and discrepancies are even higher compared to underdeveloped countries (39). Moreover, national health systems show high diversity regarding the characteristics of healthcare funding, coverage, management, and models. Hence, a significant limitation of the present review is the impossibility of generalizing the findings to the reality of low-middle-income countries. Therefore, further studies must be performed in these countries to collect representative data on the impact of home care on financially vulnerable countries.

Some countries do not have universal healthcare (such as the United States and Switzerland), some offer healthcare through partnerships with private institutions (such as Australia), and in other countries, citizens are obliged to acquire health insurance (such as Germany, the Netherlands, and Israel). Also, countries like Brazil guarantee access to comprehensive treatment for the population, including home care (40). New economies must consider the characteristics of national health systems, especially the budget source and scope of hospital and home care coverage.

The limitations of this study predominantly refer to the lack of standardization of the methodological designs of the eligible studies. Ideally, treatments would be compared using a meta-analysis, but the heterogeneity of the included studies (lack of standardization in disease stages, different cost perspectives, and diverse care approaches applied to home care) made the grouping of results impossible.

Regarding the methodology of primary studies, despite the guidelines with best practice recommendations to conduct and report cost-effectiveness in economic evaluations (41, 42), most studies did not perform them. Hence, researchers should use these guidelines and journals should promote them for standardizing economic evaluations. Although the methodological quality of the included studies was satisfactory overall, there were still considerable flaws. Moreover, the present review covers only outcomes from developed countries, whereas a global scope would be optimal, including the weaknesses and strengths of each system.

## 5 Conclusion

The data of the qualitative synthesis allow concluding that home care presents lower costs than hospital care for COPD patients. Regarding effectiveness, there is no possibility of choosing a more effective care for COPD patients, given the incipience of the data presented on eligible studies. Home care for COPD patients could be considered an alternative when managing healthcare systems, but effectiveness assessments must be carried out with vigor in future studies and evaluations. Furthermore, considering the analyzed data refer only to high-income countries, caution is required when extrapolating this conclusion to low- and low-middle-income countries.

## Data Availability

The original contributions presented in this study are included in this article/Supplementary material, further inquiries can be directed to the corresponding author.

## Footnotes

1
http://www.crd.york.ac.uk/PROSPERO

## References

[1] World Health Organization [WHO]. *Chronic obstructive pulmonary disease (COPD).* Geneva: World Health Organization [WHO] (2022).

[2] Zhu B Wang Y Ming J Chen W Zhang L. Disease burden of COPD in China: A systematic review. *Int J Chron Obstruct Pulmon Dis.* (2018) 13:1353–64. 10.2147/COPD.S161555 29731623 PMC5927339

[3] Adeloye D Song P Zhu Y Campbell H Sheikh A Rudan I Global, regional, and national prevalence of, and risk factors for, chronic obstructive pulmonary disease (COPD) in 2019: A systematic review and modelling analysis. *Lancet Respir Med.* (2022) 10:447–58. 10.1016/S2213-2600(21)00511-7 35279265 PMC9050565

[4] Sampaio M Vieira W Bernardino ÍM Herval ÁM Flores-Mir C Paranhos LR. Chronic obstructive pulmonary disease as a risk factor for suicide: A systematic review and meta-analysis. *Respir Med.* (2019) 151:11–8. 10.1016/j.rmed.2019.03.018 31047105

[5] Varmaghani M Dehghani M Heidari E Sharifi F Moghaddam S Farzadfar F. Global prevalence of chronic obstructive pulmonary disease: Systematic review and meta-analysis. *East Mediterr Health J.* (2019) 25:47–57. 10.26719/emhj.18.014 30919925

[6] Liu S Zhao Q Li W Zhao X Li K. The cost-effectiveness of pulmonary rehabilitation for copd in different settings: A systematic review. *Appl Health Econ Health Policy.* (2021) 19:313–24. 10.1007/s40258-020-00613-5 33079374

[7] Gutiérrez Villegas C Paz-Zulueta M Herrero-Montes M Parás-Bravo P Madrazo Pérez M. Cost analysis of chronic obstructive pulmonary disease (COPD): A systematic review. *Health Econ Rev.* (2021) 11:31. 10.1186/s13561-021-00329-9 34403023 PMC8369716

[8] Achelrod D Schreyögg J Stargardt T. Health-economic evaluation of home telemonitoring for COPD in Germany: Evidence from a large population-based cohort. *Eur J Health Econ.* (2017) 18:869–82. 10.1007/s10198-016-0834-x 27699567 PMC5533837

[9] Jacobs JM Marcus EL Stessman J. Prolonged mechanical ventilation: A comparison of patients treated at home compared with hospital long-term care. *J Am Med Dir Assoc.* (2021) 22:418–24. 10.1016/j.jamda.2020.06.038 32727692

[10] Helgheim BI Sandbaek B. Who is doing what in home care services? *Int J Environ Res Public Health.* (2021) 18:10504. 10.3390/ijerph181910504 34639804 PMC8508197

[11] Widyastuti K Makhabah D Setijadi A Sutanto Y Ambrosino N. Benefits and costs of home pedometer assisted physical activity in patients with COPD. A preliminary randomized controlled trial. *Pulmonology.* (2018) 24:211–8. 10.1016/j.pulmoe.2018.01.006 30008335

[12] Goossens L Utens C Smeenk F van Schayck O van Vliet M van Litsenburg W Cost-effectiveness of early assisted discharge for COPD exacerbations in The Netherlands. *Value Health.* (2013) 16:517–28. 10.1016/j.jval.2013.01.010 23796285

[13] Gallagher A Shersher V Mortimer D Truby H Haines T. The cost-effectiveness of adjunctive lifestyle interventions for the management of cancer: A systematic review. *Appl Health Econ Health Policy.* (2022) 21:225–42. 10.1007/s40258-022-00759-4 36163450 PMC9931860

[14] Grustam A Severens J De Massari D Buyukkaramikli N Koymans R Vrijhoef H. Cost-effectiveness analysis in telehealth: A comparison between home telemonitoring, nurse telephone support, and usual care in chronic heart failure management. *Value Health.* (2018) 21:772–82. 10.1016/j.jval.2017.11.011 30005749

[15] Oksman E Linna M Hörhammer I Lammintakanen J Talja M. Cost-effectiveness analysis for a tele-based health coaching program for chronic disease in primary care. *BMC Health Serv Res.* (2017) 17:138. 10.1186/s12913-017-2088-4 28202032 PMC5312514

[16] Moalosi G Floyd K Phatshwane J Moeti T Binkin N Kenyon T. Cost-effectiveness of home-based care versus hospital care for chronically ill tuberculosis patients, Francistown, Botswana. *Int J Tuberc Lung Dis.* (2003) 7:S80–5. 12971658

[17] McCarroll Z Townson J Pickles T Gregory J Playle R Robling M Cost-effectiveness of home versus hospital management of children at onset of type 1 diabetes: The DECIDE randomised controlled trial. *BMJ Open.* (2021) 11:e043523. 10.1136/bmjopen-2020-043523 34011587 PMC8137197

[18] Bakerly N Davies C Dyer M Dhillon P. Cost analysis of an integrated care model in the management of acute exacerbations of chronic obstructive pulmonary disease. *Chron Respir Dis.* (2009) 6:201–8. 10.1177/1479972309104279 19729444

[19] Shamseer L Moher D Clarke M Ghersi D Liberati A Petticrew M Preferred reporting items for systematic review and meta-analysis protocols (PRISMA-P) 2015: Elaboration and explanation. *BMJ.* (2015) 350:g7647. 10.1136/bmj.g7647 25555855

[20] Page M McKenzie J Bossuyt P Boutron I Hoffmann T Mulrow C The PRISMA 2020 statement: An updated guideline for reporting systematic reviews. *BMJ.* (2021) 372:n71. 10.1136/bmj.n71 33782057 PMC8005924

[21] Aromataris E Munn Z. *JBI manual for evidence synthesis.* JBI (2020). Available online at: https://jbi-global-wiki.refined.site/space/MANUAL (accessed March 12, 2024).

[22] Ouzzani M Hammady H Fedorowicz Z Elmagarmid A. Rayyan-a web and mobile app for systematic reviews. *Syst Rev.* (2016) 5:210. 10.1186/s13643-016-0384-4 27919275 PMC5139140

[23] Gomersall J Jadotte Y Xue Y Lockwood S Riddle D Preda A. Conducting systematic reviews of economic evaluations. *Int J Evid Based Healthc.* (2015) 13:170–8. 10.1097/XEB.0000000000000063 26288063

[24] Bergner M Hudson L Conrad D Patmont C McDonald G Perrin E The cost and efficacy of home care for patients with chronic lung disease. *Med Care.* (1988) 26:566–79. 10.1097/00005650-198806000-00005 3379988

[25] Shepperd S Harwood D Gray A Vessey M Morgan P. Randomised controlled trial comparing hospital at home care with inpatient hospital care. II: Cost minimisation analysis. *BMJ.* (1998) 316:1791–6. 10.1136/bmj.316.7147.1791 9624069 PMC28579

[26] Nicholson C Bowler S Jackson C Schollay D Tweeddale M O’Rourke P. Cost comparison of hospital- and home-based treatment models for acute chronic obstructive pulmonary disease. *Aust Health Rev.* (2001) 24:181–7. 10.1071/ah010181 11842709

[27] Puig-Junoy J Casas A Font-Planells J Escarrabill J Hernández C Alonso J The impact of home hospitalization on healthcare costs of exacerbations in COPD patients. *Eur J Health Econ.* (2007) 8:325–32. 10.1007/s10198-006-0029-y 17221178

[28] Aimonino N Tibaldi V Leff B Scarafiotti C Marinello R Zanocchi M Substitutive “hospital at home” versus inpatient care for elderly patients with exacerbations of chronic obstructive pulmonary disease: A prospective randomized, controlled trial. *J Am Geriatr Soc.* (2008) 56:493–500. 10.1111/j.1532-5415.2007.01562.x 18179503

[29] Vilà A Villegas E Cruanyes J Delgado R Sabaté R Ortega J Cost-effectiveness of a Barcelona home care program for individuals with multimorbidity. *J Am Geriatr Soc.* (2015) 63:1017–24. 10.1111/jgs.13396 25940863

[30] Brian Cassel J Kerr K McClish D Skoro N Johnson S Wanke C Effect of a home-based palliative care program on healthcare use and costs. *J Am Geriatr Soc.* (2016) 64:2288–95. 10.1111/jgs.14354 27590922 PMC5118096

[31] Soriano J García-Río F Vázquez-Espinosa E Conforto J Hernando-Sanz A López-Yepes L A multicentre, randomized controlled trial of telehealth for the management of COPD. *Respir Med.* (2018) 144:74–81. 10.1016/j.rmed.2018.10.008 30366588

[32] Duiverman M Vonk J Bladder G van Melle J Nieuwenhuis J Hazenberg A Home initiation of chronic non-invasive ventilation in COPD patients with chronic hypercapnic respiratory failure: A randomised controlled trial. *Thorax.* (2020) 75:244–52. 10.1136/thoraxjnl-2019-213303 31484786 PMC7063397

[33] Centers for Disease Control and Prevention [CDC]. *COVID data tracker.* Atlanta, GA: Centers for Disease Control and Prevention [CDC] (2022).

[34] Sandhu P Shah A Ahmad F Kerr J Demeke H Graeden E Emergency department and intensive care unit overcrowding and ventilator shortages in us hospitals during the COVID-19 pandemic, 2020-2021. *Public Health Rep.* (2022) 137:796–802. 10.1177/00333549221091781 35642664 PMC9257510

[35] Munjal S Munjal S Gao J Venketaraman V. Exploring potential COPD immunosuppression pathways causing increased susceptibility for MAC infections among COPD patients. *Clin Pract.* (2021) 11:619–30. 10.3390/clinpract11030077 34563006 PMC8482292

[36] Pinto L Alghamdi M Benedetti A Zaihra T Landry T Bourbeau J. Derivation and validation of clinical phenotypes for COPD: A systematic review. *Respir Res.* (2015) 16:50. 10.1186/s12931-015-0208-4 25928208 PMC4460884

[37] Esteban C Arostegui I Aburto M Moraza J Quintana J García-Loizaga A Chronic obstructive pulmonary disease subtypes. Transitions over time. *PLoS One.* (2016) 11:e0161710. 10.1371/journal.pone.0161710 27611911 PMC5017635

[38] Naresh B Reddy BS. Challenges and opportunity of e-learning in developed and developing countries- a review. *Int J Emerg Res Manag Tech.* (2015) 4:259–62.

[39] Frost LJ Reich MR. Creating access to health technologies in poor countries. *Health Affairs.* (2009) 28:962–73. 19597194 10.1377/hlthaff.28.4.962

[40] Ministério da Saúde. *Departamento de Atenção Básica. Caderno de atenção domiciliar. Ministério da Saúde, Secretaria de Atenção à Saúde, Departamento de Atenção Básica.* Brasília: Ministério da Saúde (2012).

[41] Evers S Goossens M de Vet H van Tulder M Ament A. Criteria list for assessment of methodological quality of economic evaluations: Consensus on health economic criteria. *Int J Technol Assess Health Care.* (2005) 21:240–5. 15921065

[42] Husereau D Drummond M Augustovski F de Bekker-Grob E Briggs A Carswell C Consolidated health economic evaluation reporting standards 2022 (CHEERS 2022) statement: Updated reporting guidance for health economic evaluations. *BMC Med.* (2022) 20:23. 10.1186/s12916-021-02204-0 35022047 PMC8753858

