# Supplementary material for: Cost-effectiveness of home care compared to hospital care in patients with chronic obstructive pulmonary disease (COPD): a systematic review
Source: Front Med (Lausanne). 2024 Oct 3;11:1405840. doi: 10.3389/fmed.2024.1405840 (PMC11484625; doi:10.3389/fmed.2024.1405840)
Supplement: Supplementary file 1 [file Data_Sheet_1.docx]

**Supplementary material 1.** Studies excluded after full-text readings and the reasons for exclusion, and studies with full texts not found (n=124).

| **Authors, year** | **Reasons for exclusion** |
| --- | --- |
| Gersten et al., 1968 | It does not assess COPD |
| Cherniack et al., 1969 | Conference abstract |
| Creek, 1982 | Conference abstract |
| Motwani and Herring, 1988 | It does not include a control group (hospital care) |
| Haggerty et al., 1991 | It does not include a control group (hospital care) |
| Williams et al., 1991 | It does not include a control group (hospital care) |
| Melin et al., 1993 | It does not include a control group (hospital care) |
| Goldberg and Trubitt, 1994 | Conference abstract |
| Opit, 1997 | It does not assess COPD |
| Bertrand et al., 1998 | Literature review |
| Güell et al., 1998 | It does not include a control group (hospital care) |
| Carrera et al., 1999 | It does not include a control group (hospital care) |
| Smith et al., 1999 | It does not include a control group (hospital care) |
| Gutteridge et al., 2000 | Conference abstract |
| Hughes et al., 2000 | It does not include a control group (hospital care) |
| Ward et al., 2000 | It does not include a control group (hospital care) |
| Zinman et al., 2000 | It does not compare home and hospital care |
| Farrero et al., 2001 | It does not include a control group (hospital care) |
| Maquilon et al., 2001 | It does not include a control group (hospital care) |
| Miravitlles and Figueras, 2001 | Literature review |
| Andersson et al., 2002 | It does not include a control group (hospital care) |
| Bernier, 2002 | Editorial |
| Gallefoss and Bakke, 2002 | It does not include a control group (hospital care) |
| Hernandez et al., 2003 | It does not include a control group (hospital care) |
| Levine et al., 2003 | Literature review |
| Neff et al., 2003 | It does not include a control group (hospital care) |
| Tuggey et al., 2003 | It does not include a control group (hospital care) |
| Golmohammadi et al., 2004 | It does not compare home and hospital care |
| Greenough et al., 2004 | It does not include a control group (hospital care) |
| Monninkhof et al., 2004 | It does not include a control group (hospital care) |
| Nishimura and Zaher, 2004 | Literature review |
| Noel et al., 2004 | It does not include a control group (hospital care) |
| Reisfield and Wilson, 2004 | Case report |
| Enguidanos et al., 2005 | It does not include a control group (hospital care) |
| Leff et al., 2005 | It does not include a control group (hospital care) |
| O'Neill et al., 2005 | It does not include a control group (hospital care) |
| Di Re et al., 2006 | It does not include a control group (hospital care) |
| Finkelstein et al., 2006 | It does not include a control group (hospital care) |
| Paré et al., 2006 | It does not include a control group (hospital care) |
| Pearson et al., 2006 | It does not include a control group (hospital care) |
| Jones et al., 2007 | It does not include a control group (hospital care) |
| Collins, 2008 | Conference abstract |
| Bendixen et al., 2009 | It does not assess COPD |
| Clini et al., 2009 | It does not include a control group (hospital care) |
| Effing et al., 2009 | It does not include a control group (hospital care) |
| Frick et al., 2009 | It does not include a control group (hospital care) |
| Hebert, 2009 | Editorial |
| Pinnock et al., 2009 | It does not include a control group (hospital care) |
| Vitacca et al., 2009 | It does not include a control group (hospital care) |
| Easley et al., 2010 | Editorial |
| Jansson et al., 2010 | It does not include a control group (hospital care) |
| Landers, 2010 | Editorial |
| Ramani et al., 2010 | It does not include a control group (hospital care) |
| Aanesen et al., 2011 | It does not assess COPD |
| Chuang et al., 2011 | It does not include a control group (hospital care) |
| Khdour et al., 2011 | It does not include a control group (hospital care) |
| Munhoz et al., 2011 | It does not include a control group (hospital care) |
| Reyes et al., 2011 | Literature review |
| Romain et al., 2011 | It does not include a control group (hospital care) |
| Vitacca et al., 2011 | It does not include a control group (hospital care) |
| Antoniu, 2012 | It does not compare home and hospital care |
| Carter, 2012 | Editorial |
| Chandra et al., 2012 | It does not include a control group (hospital care) |
| Dinesen et al., 2012 | It does not include a control group (hospital care) |
| Haesum et al., 2012 | It does not include a control group (hospital care) |
| Levine et al., 2012 | It does not include a control group (hospital care) |
| Spaulding et al., 2012 | Editorial |
| Watkins et al., 2012 | It does not include a control group (hospital care) |
| Holland et al., 2013 | Study protocol |
| Jakobsen et al., 2013 | Study protocol |
| Klinger et al., 2013 | It does not assess COPD |
| Kuwornu et al., 2013 | It does not include a control group (hospital care) |
| Liu et al., 2013 | It does not include a control group (hospital care) |
| Paré et al., 2013a | It does not include a control group (hospital care) |
| Paré et al., 2013b | It does not include a control group (hospital care) |
| D'Souza et al., 2014 | It does not compare home and hospital care |
| Edes et al., 2014 | It does not include a control group (hospital care) |
| Hazenberg et al., 2014 | It does not assess COPD |
| Jódar-Sanchez et al., 2014 | It does not include a control group (hospital care) |
| Matsumura et al., 2014 | It does not include a control group (hospital care) |
| Xie et al., 2014 | Conference abstract |
| Li et al., 2015 | It does not include a control group (hospital care) |
| McDowell et al., 2015 | It does not include a control group (hospital care) |
| Stoddart et al., 2015 | It does not include a control group (hospital care) |
| Alhelali et al., 2016 | It does not include a control group (hospital care) |
| Coughlin et al., 2016 | Conference abstract |
| Hofer et al., 2016 | It does not include a control group (hospital care) |
| Waye et al., 2016 | Conference abstract |
| Zwerink et al., 2016 | It does not include a control group (hospital care) |
| Bourdin and Molinari, 2017 | Editorial |
| Coughlin et al., 2017 | It does not include a control group (hospital care) |
| Garner et al., 2017 | It does not compare home and hospital care |
| Sorensen et al., 2017 | It does not include a control group (hospital care) |
| Udsen et al., 2017a | It does not include a control group (hospital care) |
| Udsen et al., 2017b | It does not include a control group (hospital care) |
| Clarke et al., 2018 | It does not include a control group (hospital care) |
| Cox et al., 2018 | Pilot study |
| Criner et al., 2018 | Conference abstract |
| Echevarria et al., 2018 | It does not include a control group (hospital care) |
| Kerkhof et al., 2018 | Conference abstract |
| Mirón Rubio et al., 2018 | It does not include a control group (hospital care) |
| Murphy et al., 2018 | Conference abstract |
| Satici et al., 2018 | It does not include a control group (hospital care) |
| Walker et al., 2018 | It does not include a control group (hospital care) |
| Bandurska et al., 2019 | It does not assess COPD |
| Borbeau et al., 2019 | It does not include a control group (hospital care) |
| Diette et al., 2019 | Conference abstract |
| Dolidon et al., 2019 | It does not include a control group (hospital care) |
| Moayeri et al., 2019 | It does not include a control group (hospital care) |
| Akhtar et al., 2020 | It does not include a control group (hospital care) |
| Burge et al., 2020 | It does not include a control group (hospital care) |
| Persson et al., 2020 | It does not include a control group (hospital care) |
| Tanus et al., 2020 | Conference abstract |
| Atlagic et al., 2021 | It does not assess COPD |
| Cai et al., 2021 | It does not include a control group (hospital care) |
| Hall et al., 2021 | It does not include a control group (hospital care) |
| Hernandez-Quiles et al., 2021 | Preprint |
| Sorensen et al., 2021 | It does not include a control group (hospital care) |
| Verberkt et al., 2021 | It does not include a control group (hospital care) |
| Casebeer et al., 2022 | It does not include a control group (hospital care) |
| Hendriks et al., 2022 | It does not assess COPD |
| Hwang et al., 2024 | It does not include a control group (hospital care) |
| Bower et al., 2024 | It does not assess COPD |
| Farmakis et al., 2024 | It does not include a control group (hospital care) |

**REFERENCES**

Gersten JW, Miller B, Cenkovich F, Dinken H. Comparison of home and clinic rehabilitation for chronically ill and physically disabled persons. Arch Phys Med Rehabil. 1968;49(11):615-42.

Cherniack RM, Handford RG, Svanhill E. Home care of chronic respiratory disease. Md State Med J. 1969;18(10):87-8.

Creek LV. A homecare hospice profile: description, evaluation, and cost analysis. J Fam Pract. 1982;14(1):53-8.

Motwani JK, Herring GM. Home care for ventilator-dependent persons: a cost-effective, humane public policy. Health Soc Work. 1988;13(1):20-4. <https://doi.org/10.1093/hsw/13.1.20>

Haggerty MC, Stockdale-Woolley R, Nair S. Respi-Care. An innovative home care program for the patient with chronic obstructive pulmonary disease. Chest. 1991;100(3):607-12. <https://doi.org/10.1378/chest.100.3.607>

Williams B. Comparison of services among different types of home health agencies. Med Care. 1991;32(11):1134-52.

Melin AL, Håkansson S, Bygren LO. The cost-effectiveness of rehabilitation in the home: a study of Swedish elderly. Am J Public Health. 1993;83(3):356-62. <https://doi.org/10.2105/ajph.83.3.356>

Goldberg AI, Trubitt MJ. An integrated approach to home health care. Phys Exec. 1994;20(1):45.

Opit LJ. Domiciliary care for the elderly sick--economy or neglect? Br Med J. 1977;1(6052):30-3. <https://doi.org/10.1136/bmj.1.6052.30>

Bertrand P, Alvarez C, Fabres J, Simonetti M, Sánchez I. Oxigenoterapia domiciliaria en niños con insuficiencia respiratoria crónica [Home oxygen therapy in children with chronic respiratory failure]. Rev Med Chil. 1998;126(3):284-92.

Güell R, González A, Morante F, Sangenis M, Sotomayor C, Caballero C, Sanchís J. Mejor en casa: un programa de asistencia continuada para los pacientes con enfermedad respiratoria crónica avanzada. Arch Bronconeumol. 1998;34(11):541-6. <https://doi.org/10.1016/s0300-2896(15)30336-7>

Carrera M, Sauleda J, Bauzá F, Bosch M, Togores B, Barbé F, Agustí AG. Resultados de la actuación de una unidad de control de la oxigenoterapia domiciliaria. Arch Bronconeumol. 1999;35(1):33-8. <https://doi.org/10.1016/s0300-2896(15)30322-7>

Smith BJ, Appleton SL, Bennett PW, Roberts GC, Del Fante P, Adams R, Trott CM, Allan DP, Southcott AM, Ruffin RE. The effect of a respiratory home nurse intervention in patients with chronic obstructive pulmonary disease (COPD). Aust N Z J Med. 1999;29(5):718-25. <https://doi.org/10.1111/j.1445-5994.1999.tb01621.x>

Gutteridge C, Chaytor H, King B, Goldman JM. Torbay Hospital Outreach Respiratory Team (THORT) - Hospital at home for COPD in a semi-rural setting. Thorax. 2000;55:A42.

Hughes SL, Weaver FM, Giobbie-Hurder A, Manheim L, Henderson W, Kubal JD, Ulasevich A, Cummings J; Department of Veterans Affairs Cooperative Study Group on Home-Based Primary Care. Effectiveness of team-managed home-based primary care: a randomized multicenter trial. JAMA. 2000;284(22):2877-85. <https://doi.org/10.1001/jama.284.22.2877>

Ward MM, Javitz HS, Smith WM, Bakst A. Direct medical cost of chronic obstructive pulmonary disease in the U.S.A. Respir Med. 2000;94(11):1123-9. <https://doi.org/10.1053/rmed.2000.0933>

Zinman C, Richards GA, Taylor R, Mer M. Long-term domiciliary oxygen therapy--the Johannesburg Hospital experience. S Afr Med J. 2000;90(6):617-21.

Farrero E, Escarrabill J, Prats E, Maderal M, Manresa F. Impact of a hospital-based home-care program on the management of COPD patients receiving long-term oxygen therapy. Chest. 2001;119(2):364-9. <https://doi.org/10.1378/chest.119.2.364>

Maquilón C, Chiong H, Bello S, Naranjo C, Lira P, Díaz M. Estudio comparativo de costos anuales en salud entre usuarios de oxígeno domiciliario y pacientes en lista de espera. Rev Med Chil. 2001;129(12):1395-403.

Miravitlles M, Figueras M. El coste de la enfermedad pulmonar obstructiva crónica en España. Opciones para una optimización de recursos. Arch Bronconeumol. 2001;37(9):388-93.

Andersson F, Borg S, Jansson SA, Jonsson AC, Ericsson A, Prütz C, Rönmark E, Lundbäck B. The costs of exacerbations in chronic obstructive pulmonary disease (COPD). Respir Med. 2002;96(9):700-8. <https://doi.org/10.1053/rmed.2002.1334>

Bernier L. Assessing respiratory status from a distance. Home Healthc Nurse. 2001;19(10):632-40. <https://doi.org/10.1097/00004045-200110000-00015>

Gallefoss F, Bakke PS. Cost-benefit and cost-effectiveness analysis of self-management in patients with COPD--a 1-year follow-up randomized, controlled trial. Respir Med. 2002;96(6):424-31. <https://doi.org/10.1053/rmed.2002.1293>

Hernandez C, Casas A, Escarrabill J, Alonso J, Puig-Junoy J, Farrero E, Vilagut G, Collvinent B, Rodriguez-Roisin R, Roca J; CHRONIC project. Home hospitalisation of exacerbated chronic obstructive pulmonary disease patients. Eur Respir J. 2003;21(1):58-67. <https://doi.org/10.1183/09031936.03.00015603>

Levine SA, Boal J, Boling PA. Home care. JAMA. 2003;290(9):1203-7. <https://doi.org/10.1001/jama.290.9.1203>

Neff DF, Madigan E, Narsavage G. APN-directed transitional home care model: achieving positive outcomes for patients with COPD. Home Healthc Nurse. 2003;21(8):543-50. <https://doi.org/10.1097/00004045-200308000-00010>

Tuggey JM, Plant PK, Elliott MW. Domiciliary non-invasive ventilation for recurrent acidotic exacerbations of COPD: an economic analysis. Thorax. 2003;58(10):867-71. <https://doi.org/10.1136/thorax.58.10.867>

Golmohammadi K, Jacobs P, Sin DD. Economic evaluation of a community-based pulmonary rehabilitation program for chronic obstructive pulmonary disease. Lung. 2004;182(3):187-96. <https://doi.org/10.1007/s00408-004-3110-2>

Greenough A, Alexander J, Burgess S, Chetcuti PA, Cox S, Lenney W, Turnbull F, Shaw NJ, Woods A, Boorman J, Coles S, Turner J. High versus restricted use of home oxygen therapy, health care utilisation and the cost of care in chronic lung disease infants. Eur J Pediatr. 2004;163(6):292-6. <https://doi.org/10.1007/s00431-004-1415-z>

Monninkhof E, van der Valk P, Schermer T, van der Palen J, van Herwaarden C, Zielhuis G. Economic evaluation of a comprehensive self-management programme in patients with moderate to severe chronic obstructive pulmonary disease. Chron Respir Dis. 2004;1(1):7-16. <https://doi.org/10.1191/1479972304cd005oa>

Nishimura S, Zaher C. Cost impact of COPD in Japan: opportunities and challenges? Respirology. 2004;9(4):466-73. <https://doi.org/10.1111/j.1440-1843.2004.00617.x>

Noel HC, Vogel DC, Erdos JJ, Cornwall D, Levin F. Home telehealth reduces healthcare costs. Telemed J E Health. 2004;10(2):170-83. <https://doi.org/10.1089/tmj.2004.10.170>

Reisfield GM, Wilson GR. The cost of breathing: an economic analysis of the patient cost of home oxygen therapy. Am J Hosp Palliat Care. 2004;21(5):348-52. <https://doi.org/10.1177/104990910402100508>

Enguidanos SM, Cherin D, Brumley R. Home-based palliative care study: site of death, and costs of medical care for patients with congestive heart failure, chronic obstructive pulmonary disease, and cancer. J Soc Work End Life Palliat Care. 2005;1(3):37-56. <https://doi.org/10.1300/j457v01n03_04>

Leff B, Burton L, Mader SL, Naughton B, Burl J, Inouye SK, Greenough WB 3rd, Guido S, Langston C, Frick KD, Steinwachs D, Burton JR. Hospital at home: feasibility and outcomes of a program to provide hospital-level care at home for acutely ill older patients. Ann Intern Med. 2005;143(11):798-808. <https://doi.org/10.7326/0003-4819-143-11-200512060-00008>

O'Neill B, Bradley JM, Heaney L, O'Neill C, MacMahon J. Short burst oxygen therapy in chronic obstructive pulmonary disease: a patient survey and cost analysis. Int J Clin Pract. 2005;59(7):751-3. <https://doi.org/10.1111/j.1368-5031.2005.00574.x>

Di Re L, Orsini A, Ferron F. Home long term oxygen therapy: Preliminary analysis of a service with a high degree of complexity and impact on hospitalization. Rassegna di Patologia dell'Apparato Respiratorio. 2006;21(4):181-187.

Finkelstein SM, Speedie SM, Potthoff S. Home telehealth improves clinical outcomes at lower cost for home healthcare. Telemed J E Health. 2006;12(2):128-36. <https://doi.org/10.1089/tmj.2006.12.128>

Paré G, Sicotte C, St-Jules D, Gauthier R. Cost-minimization analysis of a telehomecare program for patients with chronic obstructive pulmonary disease. Telemed J E Health. 2006;12(2):114-21. <https://doi.org/10.1089/tmj.2006.12.114>

Pearson S, Inglis SC, McLennan SN, Brennan L, Russell M, Wilkinson D, Thompson DR, Stewart S. Prolonged effects of a home-based intervention in patients with chronic illness. Arch Intern Med. 2006;166(6):645-50. <https://doi.org/10.1001/archinte.166.6.645>

Jones A, Wood-Baker R, Walters EH. Domiciliary oxygen therapy services in Tasmania: prescription, usage and impact of a specialist clinic. Med J Aust. 2007;186(12):632-4. <https://doi.org/10.5694/j.1326-5377.2007.tb01081.x>

Collins EG, Langbein WE, Fehr L, O'Connell S, Jelinek C, Hagarty E, Edwards L, Reda D, Tobin MJ, Laghi F. Can ventilation-feedback training augment exercise tolerance in patients with chronic obstructive pulmonary disease? Am J Respir Crit Care Med. 2008;177(8):844-52. <https://doi.org/10.1164/rccm.200703-477oc>

Bendixen RM, Levy CE, Olive ES, Kobb RF, Mann WC. Cost effectiveness of a telerehabilitation program to support chronically ill and disabled elders in their homes. Telemed J E Health. 2009;15(1):31-8. <https://doi.org/10.1089/tmj.2008.0046>

Clini EM, Magni G, Crisafulli E, Viaggi S, Ambrosino N. Home non-invasive mechanical ventilation and long-term oxygen therapy in stable hypercapnic chronic obstructive pulmonary disease patients: comparison of costs. Respiration. 2009;77(1):44-50. <https://doi.org/10.1159/000127410>

Effing T, Kerstjens H, van der Valk P, Zielhuis G, van der Palen J. (Cost)-effectiveness of self-treatment of exacerbations on the severity of exacerbations in patients with COPD: the COPE II study. Thorax. 2009;64(11):956-62. <https://doi.org/10.1136/thx.2008.112243>

Frick KD, Burton LC, Clark R, Mader SI, Naughton WB, Burl JB, Greenough WB, Steinwachs DM, Leff B. Substitutive Hospital at Home for older persons: effects on costs. Am J Manag Care. 2009;15(1):49-56.

Hébert R. Home care: from adequate funding to integration of services. Healthc Pap. 2009;10(1):58-64. <https://doi.org/10.12927/hcpap.2009.21224>

Pinnock H, Hanley J, Lewis S, MacNee W, Pagliari C, van der Pol M, Sheikh A, McKinstry B; TELESCOT Programme Group. The impact of a telemetric chronic obstructive pulmonary disease monitoring service: randomised controlled trial with economic evaluation and nested qualitative study. Prim Care Respir J. 2009;18(3):233-5. <https://doi.org/10.4104/pcrj.2009.00040>

Vitacca M, Bianchi L, Guerra A, Fracchia C, Spanevello A, Balbi B, Scalvini S. Tele-assistance in chronic respiratory failure patients: a randomised clinical trial. Eur Respir J. 2009;33(2):411-8. <https://doi.org/10.1183/09031936.00005608>

Easley C, Petersen R, Holmes M. The health and economic burden of chronic diseases in North Carolina. N C Med J. 2010;71(1):92-5.

Jansson SA, Stenling A, Backman H, Ronmark E, Lindberg A, Lundback B. Health care costs of individuals with and without copd in Sweden. Value in Health. 2010;13(7):1. <http://dx.doi.org/10.1016/S1098-3015(11)72250-1>

Landers SH. Why health care is going home. N Engl J Med. 2010;363(18):1690-1. <https://doi.org/10.1056/nejmp1000401>

Ramani AA, Pickston AA, Clark JL, Clark CA, Brown M. Role of the management pathway in the care of advanced COPD patients in their own homes. Care Manag J. 2010;11(4):249-53. <https://doi.org/10.1891/1521-0987.11.4.249>

Aanesen M, Lotherington AT, Olsen F. Smarter elder care? A cost-effectiveness analysis of implementing technology in elder care. Health Informatics J. 2011;17(3):161-72. <https://doi.org/10.1177/1460458211409716>

Chuang C, Levine SH, Rich J. Enhancing cost-effective care with a patient-centric chronic obstructive pulmonary disease program. Popul Health Manag. 2011;14(3):133-6. <https://doi.org/10.1089/pop.2010.0015>

Khdour MR, Agus AM, Kidney JC, Smyth BM, McElnay JC, Crealey GE. Cost-utility analysis of a pharmacy-led self-management programme for patients with COPD. Int J Clin Pharm. 2011;33(4):665-73. <https://doi.org/10.1007/s11096-011-9524-z>

Munhoz AS, Adde FV, Nakaie CM, Doria Filho U, Silva Filho LV, Rodrigues JC. Long-term home oxygen therapy in children and adolescents: analysis of clinical use and costs of a home care program. J Pediatr (Rio J). 2011;87(1):13-8. <https://doi.org/10.2223/jped.2050>

Reyes CG, Silva RO, Saldías FP. Cost-effectiveness of pulmonary rehabilitation in patients with chronic obstructive pulmonary disease. Rev Chile Enfermed Resp. 2011;27(2):153-8. <http://dx.doi.org/10.4067/S0717-73482011000200012>

Romain D, Bernady A, Etchamendy E, Barokas T, Pignede P. Coût des hospitalisations dues à une exacerbation de patients BPCO réhabilités à domicile. Rev Mal Respir. 2011;28(7):864-72. <https://doi.org/10.1016/j.rmr.2011.06.001>

Vitacca M, Bianchi L, Bazza A, Clini EM. Advanced COPD patients under home mechanical ventilation and/or long term oxygen therapy: Italian healthcare costs. Monaldi Arch Chest Dis. 2011;75(4):207-14. <https://doi.org/10.4081/monaldi.2011.208>

Antoniu SA. Roflumilast as add-on therapy to conventional inhalers in COPD: a cost-effectiveness analysis. J Comp Eff Res. 2012;1(4):315-7. <https://doi.org/10.2217/cer.12.31>

Carter D. A 'hospital at home' program shows good outcomes. Am J Nurs. 2012;112(9):18. <https://doi.org/10.1097/01.naj.0000418913.94913.99>

Chandra K, Blackhouse G, McCurdy BR, Bornstein M, Campbell K, Costa V, Franek J, Kaulback K, Levin L, Sehatzadeh S, Sikich N, Thabane M, Goeree R. Cost-effectiveness of interventions for chronic obstructive pulmonary disease (COPD) using an Ontario policy model. Ont Health Technol Assess Ser. 2012;12(12):1-61.

Dinesen B, Haesum LK, Soerensen N, Nielsen C, Grann O, Hejlesen O, Toft E, Ehlers L. Using preventive home monitoring to reduce hospital admission rates and reduce costs: a case study of telehealth among chronic obstructive pulmonary disease patients. J Telemed Telecare. 2012;18(4):221-5. <https://doi.org/10.1258/jtt.2012.110704>

Haesum LK, Soerensen N, Dinesen B, Nielsen C, Grann O, Hejlesen O, Toft E, Ehlers L. Cost-utility analysis of a telerehabilitation program: a case study of COPD patients. Telemed J E Health. 2012;18(9):688-92. <https://doi.org/10.1089/tmj.2011.0250>

Levine S, Steinman BA, Attaway K, Jung T, Enguidanos S. Home care program for patients at high risk of hospitalization. Am J Manag Care. 2012;18(8):e269-76.

Spaulding R, Velasquez SE, He J, Alloway GA. Hospital and emergency department resource usage: a cost analysis from a home telehealth project in Kansas. J Telemed Telecare. 2012;18(7):423-4. <https://doi.org/10.1258/jtt.2012.110517>

Watkins L, Hall C, Kring D. Hospital to home: a transition program for frail older adults. Prof Case Manag. 2012;17(3):117-23. <https://doi.org/10.1097/ncm.0b013e318243d6a7>

Holland AE, Mahal A, Hill CJ, Lee AL, Burge AT, Moore R, Nicolson C, O'Halloran P, Cox NS, Lahham A, Ndongo R, Bell E, McDonald CF. Benefits and costs of home-based pulmonary rehabilitation in chronic obstructive pulmonary disease - a multi-centre randomised controlled equivalence trial. BMC Pulm Med. 2013;13:57. <https://doi.org/10.1186/1471-2466-13-57>

Jakobsen AS, Laursen LC, Østergaard B, Rydahl-Hansen S, Phanareth KV. Hospital-admitted COPD patients treated at home using telemedicine technology in The Virtual Hospital Trial: methods of a randomized effectiveness trial. Trials. 2013;14:280. <https://doi.org/10.1186%2F1745-6215-14-280>

Klinger CA, Howell D, Marshall D, Zakus D, Brazil K, Deber RB. Resource utilization and cost analyses of home-based palliative care service provision: the Niagara West End-of-Life Shared-Care Project. Palliat Med. 2013;27(2):115-22. <https://doi.org/10.1177/0269216311433475>

Kuwornu JP, Lix L, Quail JM, Wang E, Osman M, Teare GF. A comparison of statistical models for analyzing episodes-of-care costs for chronic obstructive pulmonary disease exacerbations. Health Serv Out Res Methodol. 2013;13(2-4). <http://dx.doi.org/10.1007/s10742-013-0112-7>

Liu SX, Lee MC, Atakhorrami M, Tatousek J, McCormack M, Yung R, Hart N, White DP. Economic assessment of home-based COPD management programs. COPD. 2013;10(6):640-9. <https://doi.org/10.3109/15412555.2013.813447>

Paré G, Poba-Nzaou P, Sicotte C, Beaupré A, Lefrançois É, Nault D, Saint-Jules D. Comparing the costs of home telemonitoring and usual care of chronic obstructive pulmonary disease patients: A randomized controlled trial. Eur Res Telemed. 2013a;2(2):35-47. <https://doi.org/10.1016/j.eurtel.2013.05.001>

Paré G, Poba-Nzaou P, Sicotte C. Home telemonitoring for chronic disease management: an economic assessment. Int J Technol Assess Health Care. 2013b;29(2):155-61. <https://doi.org/10.1017/s0266462313000111>

D'Souza AO, Shah M, Dhamane AD, Dalal AA. Clinical and economic burden of COPD in a medicaid population. COPD. 2014;11(2):212-20. <https://doi.org/10.3109/15412555.2013.836168>

Edes T, Kinosian B, Vuckovic NH, Nichols LO, Becker MM, Hossain M. Better access, quality, and cost for clinically complex veterans with home-based primary care. J Am Geriatr Soc. 2014;62(10):1954-61. <https://doi.org/10.1111/jgs.13030>

Hazenberg A, Kerstjens HA, Prins SC, Vermeulen KM, Wijkstra PJ. Initiation of home mechanical ventilation at home: a randomised controlled trial of efficacy, feasibility and costs. Respir Med. 2014;108(9):1387-95. <https://doi.org/10.1016/j.rmed.2014.07.008>

Jódar-Sánchez F, Ortega F, Parra C, Gómez-Suárez C, Bonachela P, Leal S, Pérez P, Jordán A, Barrot E. Cost-utility analysis of a telehealth programme for patients with severe chronic obstructive pulmonary disease treated with long-term oxygen therapy. J Telemed Telecare. 2014;20(6):307-16. <https://doi.org/10.1177/1357633x14544421>

Matsumura T, Takarada K, Oki Y, Fujimoto Y, Kaneko H, Ohira M, Ishikawa A. Long-term Effect of Home Nursing Intervention on Cost and Healthcare Utilization for Patients with Chronic Obstructive Pulmonary Disease: A Retrospective Observational Study. Rehabil Nurs. 2015;40(6):384-9. <https://doi.org/10.1002/rnj.189>

Xie L, Kariburyo MF, Du J, Baser O. Examination of the Burden of Illness of U. S. Medicare Patients Diagnosed with Chronic Obstructive Pulmonary Disease. Value Health. 2014;17(7):A592-3. <https://doi.org/10.1016/j.jval.2014.08.2035>

Li P, Gong Y, Zeng G, Ruan L, Li G. A new mode of community continuing care service for COPD patients in China: participation of respiratory nurse specialists. Int J Clin Exp Med. 2015;8(9):15878-88.

McDowell JE, McClean S, FitzGibbon F, Tate S. A randomised clinical trial of the effectiveness of home-based health care with telemonitoring in patients with COPD. J Telemed Telecare. 2015;21(2):80-7. <https://doi.org/10.1177/1357633x14566575>

Stoddart A, van der Pol M, Pinnock H, Hanley J, McCloughan L, Todd A, Krishan A, McKinstry B. Telemonitoring for chronic obstructive pulmonary disease: a cost and cost-utility analysis of a randomised controlled trial. J Telemed Telecare. 2015;21(2):108-18. <https://doi.org/10.1177/1357633x14566574>

Alhelali RA, McNabb SJ, Memish ZA. Evaluation of home respiratory therapy delivered to patients in the Ministry of Health's Home Medical Program (HMP) and administered through the Madinah HMP Center, Kingdom of Saudi Arabia, 2013. J Epidemiol Glob Health. 2016;6(1):19-27. <https://doi.org/10.1016/j.jegh.2015.07.006>

Coughlin S, Peyerl FW, Munson SH, Ravindranath AJ, Lee-Chiong JTL. Budget impact analysis of home advanced non-invasive ventilation for severe chronic obstructive pulmonary disease. Value in Health. 2016;19(3):299. <https://doi.org/10.1016/j.jval.2016.03.690>

Hofer F, Achelrod D, Stargardt T. Cost-Utility Analysis of Telemonitoring Interventions for Patients with Chronic Obstructive Pulmonary Disease (COPD) in Germany. Appl Health Econ Health Policy. 2016;14(6):691-701. <https://doi.org/10.1007/s40258-016-0267-9>

Waye A, Jacobs P, Ospina M, Stickland M, Mayers I. Economic Surveillance For Chronic Obstructive Pulmonary Disease In Alberta, Canada. Value in Health. 2016;19(3):A14-5. <https://doi.org/10.1016/j.jval.2016.03.279>

Zwerink M, Kerstjens HA, van der Palen J, van der Valk P, Brusse-Keizer M, Zielhuis G, Effing T. (Cost-)effectiveness of self-treatment of exacerbations in patients with COPD: 2 years follow-up of a RCT. Respirology. 2016;21(3):497-503. <https://doi.org/10.1111/resp.12697>

Bourdin A, Molinari N. Quelles sont les conséquences socio-économiques des exacerbations de BPCO ? [What are the socio-economic consequences of acute COPD exacerbations?]. Rev Mal Respir. 2017;34(4):338-342. <https://doi.org/10.1016/j.rmr.2017.03.004>

Coughlin S, Peyerl FW, Munson SH, Ravindranath AJ, Lee-Chiong TL. Cost Savings from Reduced Hospitalizations with Use of Home Noninvasive Ventilation for COPD. Value Health. 2017;20(3):379-387. <https://doi.org/10.1016/j.jval.2016.09.2401>

Garner A, Hodson M, Ketsetzis G, Pulle L, Yorke J, Bhowmik A. An analysis of the economic and patient outcome impact of an integrated COPD service in east London. Int J Chron Obstruct Pulmon Dis. 2017;12:1653-1662. <https://doi.org/10.2147/copd.s127843>

Sørensen SS, Pedersen KM, Weinreich UM, Ehlers L. Economic Evaluation of Community-Based Case Management of Patients Suffering From Chronic Obstructive Pulmonary Disease. Appl Health Econ Health Policy. 2017;15(3):413-424. <https://doi.org/10.1007/s40258-016-0298-2>

Udsen WF, Lilholt PH, Hejlesen OK, Ehlers LH. Subgroup analysis of telehealthcare for patients with chronic obstructive pulmonary disease: the cluster-randomized Danish Telecare North Trial. Clinicoecon Outcomes Res. 2017a;9:391-401. <https://doi.org/10.2147/ceor.s139064>

Udsen WF, Lilholt PH, Hejlesen O, Ehlers L. Cost-effectiveness of telehealthcare to patients with chronic obstructive pulmonary disease: results from the Danish 'TeleCare North' cluster-randomised trial. BMJ Open. 2017b;7(5):e014616. <https://doi.org/10.1136/bmjopen-2016-014616>

Clarke M, Fursse J, Brown-Connolly NE, Sharma U, Jones R. Evaluation of the National Health Service (NHS) Direct Pilot Telehealth Program: Cost-Effectiveness Analysis. Telemed J E Health. 2018;24(1):67-76. <https://doi.org/10.1089/tmj.2016.0280>

Cox M, O'Connor C, Biggs K, Hind D, Bortolami O, Franklin M, Collins B, Walters S, Wailoo A, Channell J, Albert P, Freeman U, Bourke S, Steiner M, Miles J, O'Brien T, McWilliams D, Schofield T, O'Reilly J, Hughes R. The feasibility of early pulmonary rehabilitation and activity after COPD exacerbations: external pilot randomised controlled trial, qualitative case study and exploratory economic evaluation. Health Technol Assess. 2018;22(11):1-204. <https://doi.org/10.3310/hta22110>

Criner GJ, Gu Q, Murphy PB, Fusfeld L, Brueggenjuergen B, Goss T, Hart N. Cost-Effectiveness of Home Oxygen Therapy-Home Mechanical Ventilation (HOT-HMV) for Treatment of Chronic Obstructive Pulmonary Disease (COPD) with Chronic Hypercapnic Respiratory Failure Following an Acute Exacerbation of COPD in the United States (US). Am J Resp Crit Care Med. 2018;197:A2518.

Echevarria C, Gray J, Hartley T, Steer J, Miller J, Simpson AJ, Gibson GJ, Bourke SC. Home treatment of COPD exacerbation selected by DECAF score: a non-inferiority, randomised controlled trial and economic evaluation. Thorax. 2018;73(8):713-722. <https://doi.org/10.1136/thoraxjnl-2017-211197>

Kerkhof M, Rastogi S, Alacqua M, Jones R, Rhee CK, Miravitalles M, Pavord I, Price D. COPD-related health care costs for GOLD C/D patients with elevated blood eosinophil counts. Thorax. 2018;73(4):A267. <http://dx.doi.org/10.1136/thorax-2018-212555.218>

Mirón Rubio M, Ceballos Fernández R, Parras Pastor I, Palomo Iloro A, Fernández Félix BM, Medina Miralles J, Zamudio López E, González Pastor J, Amador Lorente C, Mena Hortelano N, Domínguez Sánchez A, Alonso-Viteri S. Telemonitoring and home hospitalization in patients with chronic obstructive pulmonary disease: study TELEPOC. Expert Rev Respir Med. 2018;12(4):335-343. <https://doi.org/10.1080/17476348.2018.1442214>

Murphy PB, Brueggenjuergen B, Reinhold T, Fusfeld L, Gu Q, Goss T, Hart N. Cost-effectiveness of home oxygen therapy-home mechanical ventilation (HOT-HMV) for the treatment of chronic obstructive pulmonary disease (COPD) with chronic hypercapnic respiratory failure following an acute exacerbation of COPD in the United Kingdom (UK). Am J Resp Crit Care Med. 2018;197:A2518.

Satici C, Arpinar Yigitbas B, Seker B, Demirkol MA, Kosar AF. Does Adherence to Domiciliary NIMV Decrease the Subsequent Hospitalizations Rates and Cost for Patients Diagnosed with COPD? COPD. 2018;15(3):303-309. <https://doi.org/10.1080/15412555.2018.1500532>

Walker PP, Pompilio PP, Zanaboni P, Bergmo TS, Prikk K, Malinovschi A, Montserrat JM, Middlemass J, Šonc S, Munaro G, Marušič D, Sepper R, Rosso R, Siriwardena AN, Janson C, Farré R, Calverley PMA, Dellaca' RL. Telemonitoring in Chronic Obstructive Pulmonary Disease (CHROMED). A Randomized Clinical Trial. Am J Respir Crit Care Med. 2018;198(5):620-628. <https://doi.org/10.1164/rccm.201712-2404oc>

Bandurska E, Damps-Konstańska I, Popowski P, Jędrzejczyk T, Janowiak P, Świętnicka K, Zarzeczna-Baran M, Jassem E. Cost-Effectiveness Analysis of Integrated Care in Management of Advanced Chronic Obstructive Pulmonary Disease (COPD). Med Sci Monit. 2019;25:2879-2885. <https://doi.org/10.12659/msm.913358>

Bourbeau J, Granados D, Roze S, Durand-Zaleski I, Casan P, Köhler D, Tognella S, Viejo JL, Dal Negro RW, Kessler R. Cost-effectiveness of the COPD Patient Management European Trial home-based disease management program. Int J Chron Obstruct Pulmon Dis. 2019;14:645-657. <https://doi.org/10.2147/copd.s173057>

Diette G, Craver C, Cooper L, Marquez C, Goss T. Assessment of the Impact of U.S. Medicare Competitive Bidding Program (CBP) for Home Oxygen Therapy on Medicare Program Utilization and Patient Outcomes. Am Thorac Soc. 2019;2019. <http://dx.doi.org/10.1164/ajrccm-conference.2019.199.1_MeetingAbstracts.A7114>

Dolidon S, Dupuis J, Molano Valencia LC, Salaün M, Thiberville L, Muir JF, Cuvelier A, Patout M. Characteristics and outcome of patients set up on high-flow oxygen therapy at home. Ther Adv Respir Dis. 2019;13:1753466619879794. <https://doi.org/10.1177/1753466619879794>

Moayeri F, Dunt D, Hsueh YA, Doyle C. Cost-utility analysis of telephone-based cognitive behavior therapy in chronic obstructive pulmonary disease (COPD) patients with anxiety and depression comorbidities: an application for willingness to accept concept. Expert Rev Pharmacoecon Outcomes Res. 2019;19(3):331-340. <https://doi.org/10.1080/14737167.2019.1536550>

Akhtar S, Srinivasan V, Weisse C, DiSorbo P. Characterizing the Financial Value of In-Home Palliative Care for Patients, Payers, and Hospitals. Am J Hosp Palliat Care. 2020;37(3):196-200. <https://doi.org/10.1177/1049909119872486>

Burge AT, Holland AE, McDonald CF, Abramson MJ, Hill CJ, Lee AL, Cox NS, Moore R, Nicolson C, O'Halloran P, Lahham A, Gillies R, Mahal A. Home-based pulmonary rehabilitation for COPD using minimal resources: An economic analysis. Respirology. 2020;25(2):183-190. <https://doi.org/10.1111/resp.13667>

Persson HL, Lyth J, Lind L. The Health Diary Telemonitoring and Hospital-Based Home Care Improve Quality of Life Among Elderly Multimorbid COPD and Chronic Heart Failure Subjects. Int J Chron Obstruct Pulmon Dis. 2020;15:527-541. <https://doi.org/10.2147/copd.s236192>

Tanus AF, Herrera-Rodriguez MA, Botero R, Castro C, Gómez de la Rosa F, Marrugo AC, Alvis-Gusman N. Health care costs of copd in Colombia. Value in Health. 2020;23(1):S357. <https://doi.org/10.1016/j.jval.2020.04.1369>

Atlagic FP, Parra CV, Muller NB, Flores PS, Barrios FM, Abraham MJ, Catalán NAV. Hospitalización domiciliaria en niños y adolescentes con necesidades especiales de atención en salud (NANEAS): perspectivas financieras en hospital de alta complejidad, Chile. Rev Fac Med Hum. 2021;21(4):1-10. <https://doi.org/10.25176/RFMH.v21i4.4267>

Cai S, Intrator O, Chan C, Buxbaum L, Haggerty MA, Phibbs CS, Schwab E, Kinosian B. Association of Costs and Days at Home With Transfer Hospital in Home. JAMA Netw Open. 2021;4(6):e2114920. <https://doi.org/10.1001%2Fjamanetworkopen.2021.14920>

Hall J, Turner AM, Dretzke J, Moore D, Jowett S. Cost-effectiveness of domiciliary non-invasive ventilation in patients with chronic obstructive pulmonary disease. Thorax. 2021:thoraxjnl-2021-217463. <https://doi.org/10.1136/thoraxjnl-2021-217463>

Hernandez-Quiles C, Bernabeu-Wittel M, Barón-Franco B, Palacios AA, Garcia-Serrano MR, Lopez-Jimeno W, Antonio Perez-de-Leon-Serrano J, Gómez-Barranco JM, Ruiz-Cantero A, Quero-Haro M, Cubiles-Montero E, Vergara-Lopez S, Ollero-Baturone M. A randomized clinical trial of home telemonitoring in patients with advanced heart and lung diseases. J Telemed Telecare. 2021:1357633X211059707. <https://doi.org/10.1177/1357633x211059707>

Sørensen SS, Storgaard LH, Weinreich UM. Cost-Effectiveness of Domiciliary High Flow Nasal Cannula Treatment in COPD Patients with Chronic Respiratory Failure. Clinicoecon Outcomes Res. 2021;13:553-564. <https://doi.org/10.2147/ceor.s312523>

Verberkt CA, van den Beuken-van Everdingen MHJ, Dirksen CD, Schols JMGA, Vanfleteren LEGW, Franssen FME, Groenen MTJ, Wouters EFM, Janssen DJA. Healthcare and Societal Costs in Patients with COPD and Breathlessness after Completion of a Comprehensive Rehabilitation Program. COPD. 2021;18(2):170-180. <https://doi.org/10.1080/15412555.2020.1868420>

Casebeer AW, Ronning D, Schwartz R, Long C, Bhattacharya R, Uribe C, Brown CR, Cameron J, Painter P, Sharma A, Spitale S, Powers B, Stemple C, Shrank W. A Comparison of Home Health Utilization, Outcomes, and Cost Between Medicare Advantage and Traditional Medicare. Med Care. 2022;60(1):66-74. <https://doi.org/10.1097/mlr.0000000000001661>

Hendriks SV, van den Hout WB, van Bemmel T, Bistervels IM, Eijsvogel M, Faber LM, Hofstee HMA, van der Hulle T, Iglesias Del Sol A, Kruip MJHA, Mairuhu ATA, Middeldorp S, Nijkeuter M, Huisman MV, Klok FA; YEARS Investigators. Home Treatment Compared to Initial Hospitalization in Normotensive Patients with Acute Pulmonary Embolism in the Netherlands: A Cost Analysis. Thromb Haemost. 2022 Mar;122(3):427-433.

https://doi.org/10.1055/a-1518-1847

Hwang YS, Kim WJ, Kim TH, Park Y, Jung SM, Jo HS. Cost-utility analysis of transitional care services for older inpatients with chronic obstructive pulmonary disease (COPD) in Korea. Cost Eff Resour Alloc. 2024 Mar 2;22(1):19.

<https://doi.org/10.1186/s12962-024-00526-3>

Bower KA, Hallock J, Li X, Kent T, Wardlow L. Cost and Utilization Implications of a Health Plan's Home-Based Palliative Care Program. J Palliat Med. 2024 Apr;27(4):464-470. https://doi.org/10.1089/jpm.2023.0401

Farmakis IT, Kaier K, Hobohm L, Mohr K, Valerio L, Barco S, Konstantinides SV, Binder H. Healthcare resource utilisation and associated costs after low-risk pulmonary embolism: pre-specified analysis of the Home Treatment of Pulmonary Embolism (HoT-PE) study. Clin Res Cardiol. 2024 Jan 3.

https://doi.org/10.1007/s00392-023-02355-5
